# Src Is Required for Mechanical Stretch-Induced Cardiomyocyte Hypertrophy through Angiotensin II Type 1 Receptor-Dependent β-Arrestin2 Pathways

**DOI:** 10.1371/journal.pone.0092926

**Published:** 2014-04-03

**Authors:** Shijun Wang, Hui Gong, Guoliang Jiang, Yong Ye, Jian Wu, Jieyun You, Guoping Zhang, Aijun Sun, Issei Komuro, Junbo Ge, Yunzeng Zou

**Affiliations:** 1 Shanghai Institute of Cardiovascular Diseases, Zhongshan Hospital, Fudan University, Shanghai, China; 2 Institutes of Biomedical Science, Fudan University, Shanghai, China; 3 Department of Cardiovascular Medicine, the University of Tokyo Graduate School of Medicine, Tokyo, Japan; University of Western Ontario, Canada

## Abstract

Angiotensin II (AngII) type 1 receptor (AT1-R) can be activated by mechanical stress (MS) without the involvement of AngII during the development of cardiomyocyte hypertrophy, in which G protein-independent pathways are critically involved. Although β-arrestin2-biased signaling has been speculated, little is known about how AT1-R/β-arrestin2 leads to ERK1/2 activation. Here, we present a novel mechanism by which Src kinase mediates AT1-R/β-arrestin2-dependent ERK1/2 phosphorylation in response to MS. Differing from stimulation by AngII, MS-triggered ERK1/2 phosphorylation is neither suppressed by overexpression of RGS4 (the negative regulator of the G-protein coupling signal) nor by inhibition of Gαq downstream protein kinase C (PKC) with GF109203X. The release of inositol 1,4,5-triphosphate (IP_3_) is increased by AngII but not by MS. These results collectively suggest that MS-induced ERK1/2 activation through AT1-R might be independent of G-protein coupling. Moreover, either knockdown of β-arrestin2 or overexpression of a dominant negative mutant of β-arrestin2 prevents MS-induced activation of ERK1/2. We further identifies a relationship between Src, a non-receptor tyrosine kinase and β-arrestin2 using analyses of co-immunoprecipitation and immunofluorescence after MS stimulation. Furthermore, MS-, but not AngII-induced ERK1/2 phosphorylation is attenuated by Src inhibition, which also significantly improves pressure overload-induced cardiac hypertrophy and dysfunction in mice lacking AngII. Finally, MS-induced Src activation and hypertrophic response are abolished by candesartan but not by valsartan whereas AngII-induced responses can be abrogated by both blockers. Our results suggest that Src plays a critical role in MS-induced cardiomyocyte hypertrophy through β-arrestin2-associated angiotensin II type 1 receptor signaling.

## Introduction

Angiotensin II type 1 (AT1) receptor, belonging to the G-protein-coupled receptor family (GPCRs), shares a common structure of 7-transmembrane receptor (7TMRs), and mediates the signal response of Angiotensin II (AngII), thereby regulates blood pressure, cardiac hypertrophy and heart failure [1], [2]. Recent studies demonstrate that AT1-R acts as a stress-sensitive switcher which can be triggered by mechanical stress without the ligand binding [3]–[5]. We have previously showed that some kinds of angiotensin II receptor blockers (ARBs), such as candesartan and olmesartan, inhibit pressure overload-induced cardiac hypertrophy in angiotensinogen knockout (AGT KO) mice, while others like valsartan, exert the inhibitory effect in the presence of AngII [6]. However, the detailed molecular mechanisms of how mechanical stress-induced AT1-R activation and its inhibition are regulated by ARBs still remain elucidative.

A growing body of evidences indicated that biased agonism might selectively induce the conformational switch of GPCRs, which preferentially activated or inhibited a subset of downstream signaling [7], [8]. We thus assumed there might be an unique pathway involved in mechanical stress (MS)-induced AT1-R activation and signal transduction, which was different from that induced by AngII, and resulted in the divergent effects of various ARBs. Rakesh K. previously reported that β-arrestin2 dependent pathway rather than G-protein coupling played a pivotal role in MS-induced AT1-R signaling [7]. However, little was known regarding to the downstream pathway subsequent to β-arrestin2-mediated AT1-R and ERK1/2 activation by mechanical stress. It was believed that AngII stimulated G-protein-dependent ERK1/2 activation through binding to AT1-R, but overexpression of AT1-R mutant lacking Gαq/Gαi coupling also induced ERK1/2 phosphorylation and developed severe myocardial hypertrophy both in vitro and in vivo [9], [10]. We previously reported that G protein-independent calcineurin was critically involved in mechanical stretch induced myocardial hypertrophy [11], and candesartan could attenuate the hypertrophic response through regulating the tyrosine kinases cascade [3]. Therefore, we hypothesized that Src family kinase, the non-receptor tyrosine kinase, might mediate G protein-independent AT1-R signaling and cardiac hypertrophy induced by MS.

Src is a stress-sensitive kinase which plays an important role in the pathophysiological mechanisms for pressure overload-induced myocardial hypertrophy and pulmonary arterial hypertension, Src inhibition can effectively reverse the hypertensive response and hypertrophic signaling [12], [13]. MS-mediated autophosphorylation of Src is highly associated with ERK1/2 activation [14]. Recent study revealed the expression and distribution of Src in the nucleus of cardiomyocytes with hypertrophy [15], and β-arrestin2 enhanced nuclear localization of ERK1/2 via GPCRs activation [16]. Mutant AT1 receptor lacking the docking site impaired Src-dependent nuclear translocation of ERK1/2 [10]. These findings prompted us to assume that Src kinase might be involved in β-arrestin2-meidated ERK1/2 activation and cardiac hypertrophy subsequent to AT1-R activation by MS. In the present study, we focused on the changes of AT1-R downstream signals, especially the role of Src kinase, in regulating β-arrestin2-dependent and AT1-R-induced signal transduction after MS.

## Materials and Methods

### Reagents

Anti-ERK1/2 (#9102), anti-phospho-ERK1/2 at Thr^202^/Tyr^204^ (#9101) and anti-phospho-Src at Tyr^416^ (#2101) were purchased from Cell-signaling Technology; anti-Angiotensin II type 1 receptor (ab9391) were purchased from Abcam, plc; anti-FLAG-probe (#F7425), GF109203X (#G2911) were purchased from Sigma-Aldrich; anti-HA-probe (sc-805), anti-β-arrestin2 (sc-13140) were purchased from Santa Cruz Biotechnology; and SU6656 (#572635) was purchased from Calbiochem, Merck KGaA. Mechanic stretch-model culture plates were provided as kind gifts from Chiba University Graduate School of Medicine.

### Plasmids Constructs

HA-tagged ERK2, FLAG-tagged β-arrestin1 and 2 were kindly provided by professor Issei Komuro (Tokyo University, Japan). Wild-type FLAG-tagged β-arrestin2 in vector pcDNA3 was used as the template. PCR based site-directed mutagenesis approach was performed to make a valine(54)-to-aspartic substitution in the wild-type β-arrestin2 using Pfu polymerase (Takara), the mutagenic primers were as follows: 5′-GACCGGAAAGACTTTGTGACC-3′ (forward) and 5′-GGTCACAAAGTCTTTCCGGTC-3′ (reverse). The mutant β-arrestin2-V54D was amplified and then cloned into plasmid pcDNA3. The SRC gene was amplified by PCR using primers 5′-CGGGATCCACTAGTAACGGCCGCCAG-3′ (forward) with a *BamH*I restriction site and 5′-CGCTCGAGCGAGGTTCTCTCCAGGCTG-3′ with a *Xho*I restriction site, and subcloned into the pcDNA3 plasmid containing a HA tag-encoding sequence.

### Cell Culture and transfection

In vitro cardiacmyocytes were cultured as previously reported [34]. In brief, neonatal (one-day-old) rats were sacrificed under ether anesthesia, and then ventricular tissues were surgically isolated from the anesthetized rats, all operations were made to minimize suffering. The isolated tissues were minced and placed in culture medium containing the buffers of 5.4 mmol/L KCl, 0.44 mmol/L NaH_2_PO_4_, 137 mmol/L NaCl, 4.2 mmol/L NaHCO_3_ and 5 mmol/L glucose at pH 7.4. Cells were then dissociated at 37°C by a combination of mechanical agitation and enzymatic digestion with 0.1 mg/mL DNase II (Sigma) and 0.125% pancreatin trypsin (Calbiochem). Cells were pre-plated for 2 h in 100 mm dishes with Dulbecco's modified Eagle's medium (DMEM) with 10% defined bovine calf serum (FBS, HyClone), penicillin (100 units/mL) and streptomycin (100 mg/mL) (Sigma), then the unattached cardiomyocytes were collected and plated at a field density of 1×10^5^ cells/cm^2^ on silicone rubber culture dishes. Stretching of cardiacmyocytes by 10% was conducted as described previously [3]. HEK-293-AT1 cells lines were kindly provided by professor Issei Komuro (Tokyo University, Japan), and were cultured in Dulbecco's modified Eagles medium (DMEM) with 10% FBS and 1% penicillinystreptomycin as described previously [3]. Transient transfection of AT1 plasmids into cells were performed by using Gene Transfection System (Invitrogen) according to the manufacturer's instructions. The stable selection of transfection cells were achieved by adding Aminoglycoside G418 (200 ug/mL) to cells 3 day after transfection. All cell cultures were transferred to serum-free media 24 h before experiment.

### Experimental Animal Model

Angiotensinogen gene knockout (AGT KO) mice were provided as kind gifts from professor Issei Komuro. Aged 8∼10 weeks of AGT KO mice were used in the present study and wide-type (WT) C57BL/6 mice were used as control littermates. All the procedures involving animals were carried out in accordance with the recommendations of the guidelines for the Care and Use of Laboratory Animals from China Council on Animal Care. The experiment was approved by the Experimental Animal Ethics Committee, Fudan University Shanghai Medical College with the permit number of 20110307-092. Pressure overload model was established by transverse aorta constriction (TAC) for 2 weeks as previously described [6], [11]. To ameliorate suffering, the mice were anesthetized by intraperitoneal injection of a combination of ketamine (100 mg/kg) with xylazine (5 mg/kg), and respiration was artificially controlled with a tidal volume of 0.2 ml and a respiratory rate of 110 breaths/min. The transverse aorta was constricted with the 7-0 nylon strings by ligating the aorta together with a blunted 27-gauge needle to yield a narrowing of 0.4 mm in diameter, and the needle was pulled out later. SU6656 (1 mg/kg/day) was continuously administered by Alzet osmotic mini pumps (DURECT, Cupertino, California) and was implanted subcutaneously into the back of mice right after anesthetized with 2% inhaled isoflurane from 3 days before TAC to 2 weeks after TAC. At 2 weeks after TAC, all mice were anesthetized by inhaled anesthetic isoflurane for cardiac echocardiography and hemodynamics analysis, and then mice were quickly sacrificed before they woke up. The hearts were excised for further examination, and the bodies were recorded, collected and centralized processing.

### ERK2 kinase activity Assay

HA-tagged ERK2 plasmid was transient transfected into cardiomyocytes 24 h after plating the cells on stretch-model culture plates. ERK2 kinase activity was determined as we described previously [17]. In brief, RGS4 plasmid DNA (7.5 µg) was co-transfected into each dish of HEK-293-AT1 cells with or without HA tagged-ERK2 (2.5 µg), then the transfected cells were lysed, and the lysates were incubated with anti-HA antibody for 1 h at 4°C. Then, the immunocomplex was precipitated using A/G Plus-agarose beads, washed, resuspended in 25 ml kinase buffer with 2 m Ci of [γ-^32^P] ATP, and incubated with 25 mg of MBP as the substrate at 25°C for 10 min. After incubation, the reaction was terminated by adding Laemmli sample buffer (0.002% bromphenol blue, 0.01M sodium phosphate buffer, pH 7.0, 10% glycerol, 0.4%SDS, and 1% 2-mercaptoethanol) to the samples, and the samples were boiled for 5 min. The supernatants were subjected to SDS-polyacrylamide gel electrophoresis, and the gel was washed with 7% acetic acid for 30 min and with 3% glycerol for 30 min, dried, and then subjected to autoradiography.

### RT-PCR analysis

Total RNA were extracted from cardiomyocytes using Trizol (Invitrogen) after mechanical stretch for 10 min. The cDNA was synthesized and optimized using a cDNA reverse transcription Kit (Takara), and the PCR primers used as follows:β-arrestin1, the sense primer:5′-CTGGATGTCTTGGGTCTGA-3′ and anti-sense primer: 3′-CGGATGGGGAAGTGGAAAC-5′; β-arrestin2, the sense primer: 5′-CCCTATGCTCAGAAACGAA-3′ and the anti-sense primer: 3′-TAGACAAGTAGCGGTGGAT-5′. The amplification of target genes were carried out on MJ Mini™ Gradient Thermal Cycler with a program of 95°C for 3 min followed by 35 cycles of 95°C for 30 s, 54°C for 30 s and 72°C for 30 s, and by a single incubation at 72°C for 5 min. PCR products were separated by electrophoresis on 1.2% agarose gels.

### Western blotting (WB) Analysis

Cardiomyocytes were digested and separated to mini tubes. After incubating with cell lysates for 30 min on ice, cells supernatant was obtained by centrifugating for 30 min.Total protein extracts were size-fractionated by SDS-PAGE and were transferred to PVDF membranes. The membranes were blocked with 5% FBS in TBS-T buffer (20 umol/L Tris pH 7.4, 150 mmol/L NaCl and 0.05% Tween 20) for 2 h, then were incubated with antibody at 4°C overnight. After washing them for 3 times, the second antibody was adding, finally the membranes were treated with supersignal West Pico Chemiluminescent Substrate (Thermo), and the immunoreactive bands were detected by chemiluminescence system (Bio-Rad).

### Immunoprecipitation (IP) and WB analysis

Cultured cells were transfected with various plasmids as indicated. After transfection for 24 h, cells were harvested by lysis buffer (10 mmol/L Tris-HCl, pH 7.4, 150 mmol/L NaCl, 1 mmol/L EDTA, 1% Triton X-100, 2.5 mmol/L sodium pyrophosphate, 25 mmol/L β-glycerol phosphate, 10 mmol/L NaF, 10 µg/mL aprotinin, 10 µg/mL leupeptin, 1 mmol/L PMSF). Cell extracts were sonicated and then centrifuged at 15000 g for 30 min at 4°C. Supernatants were subjected to immunoprecipitaion with indicated antibodies and co-incubated with protein A/G Plus-agarose beads (Santa Cruz Biotechnology) at 4°C for 3 h. Then the beads were spun down at 800 g for 2 min, washed with 700 µL lysis buffer for 3 times. The beads were eluted with SDS/PAGE loading buffer after extensive washed with 0.1 mol/L Tris-buffered saline. Finally the immunoprecipitates and total cell lysates were subjected to SDS/PAGE and determined by Western blotting with indicated antibodies.

### Immunocytochemistry Analysis

In vitro cultured cardiomyocytes were seeded in photic-bottomed stretch plate, and were transfected with β-arrestin2-GFP-WT or β-arrestin2-GFP-V54D mutant. Cells were stretched for 10 min, and washed with 1×PBS and fixed in 4% formaldehyde for 15 minutes at room temperature. After permeabililized with 0.1% triton X-100 for 10 minutes at room temperature, cells were blocked in 5% BSA for 1.5 hours. Immunostaining was performed by using anti-phospho-Src antibody overnight at 4°C. Oil immersion lens in a multitrack mode using a dual excitation (Alexa Fluor488 and Alexa Fluor555) filter sets. All Cells were counterstained with DAPI staining. Immunostaining was visualized by using laser scanning microscope (Leica Microsystems, Bensheim, Germany).

### Detection of Inositol Phosphates

Determination of IP_3_ was according to the methods as described previously [16]. In brief, cells were replated into 24-well plates at 1.5×10^5^ cells per well and labeled for 24 h at 37°C with myo-[^3^H] inositol (1.0 µCi/mL). After terminated labeling by aspirating the medium, cells were rinsing twice and the pellet was resuspended and harvested with phosphate-buffered saline (0.02% EDTA). Cells were transferred to culture template for re-adherence, and after subjecting to mechanical stretch or incubated with AngII (10^−7^ mol/L), cell lysates were prepared in 0.4M perchloric acid and neutralized in 0.72M KOH and 0.6M KHCO_3_ in the presence of 5 mM LiCl. The lysates were then applied to Dowex columns (AG1-X8; Bio-Rad), washed, and eluted. The eluates were counted in a liquid scintillation counter. IP_3_ releasing was estimated by determining the ratio of inositol phosphate radioactivity to the sum of total inositol phosphate.

### Echocardiography Analyses and Hemodynamic Measurements

Transthoracic echocardiography was performed by animal specific instrument (Visual Sonics® Vevo770®, VisualSonics Inc. Canada). Mice were anesthetized and M-mode images of the left ventricle were record when mice partially recovered from anesthesia. The anterior and posterior wall thickness of left ventricular (LVAWD, LVPWD) at end-diastole and papillary muscle level was measured by two-dimensional short-axis views of the left ventricle and M-mode tracings. Left ventricle ejection fraction (LVEF) was obtained using Simpson approach. Left ventricular hemodynamics was also evaluated at 2 weeks after TAC. Briefly, a micronanometer catheter (Millar 1.4F, SPR 835, Millar Instruments, Houston, TX, USA) was inserted through the right common carotid artery into the aorta and carefully introduced into left ventricular. Left ventricular end systolic pressure (LVSBP) was measured as previously described [6].

### Statistical Analysis

All data were expressed as means ± s.e.m. The between-group comparisons of means were done by one-way ANOVA followed by Tukey-Kramer test. *P* values smaller than 0.05 were considered statistical significance.

## Results

### RGS4 failed to inhibit mechanical stretch-induced ERK1/2 phosphorylation

**Figure 1:**
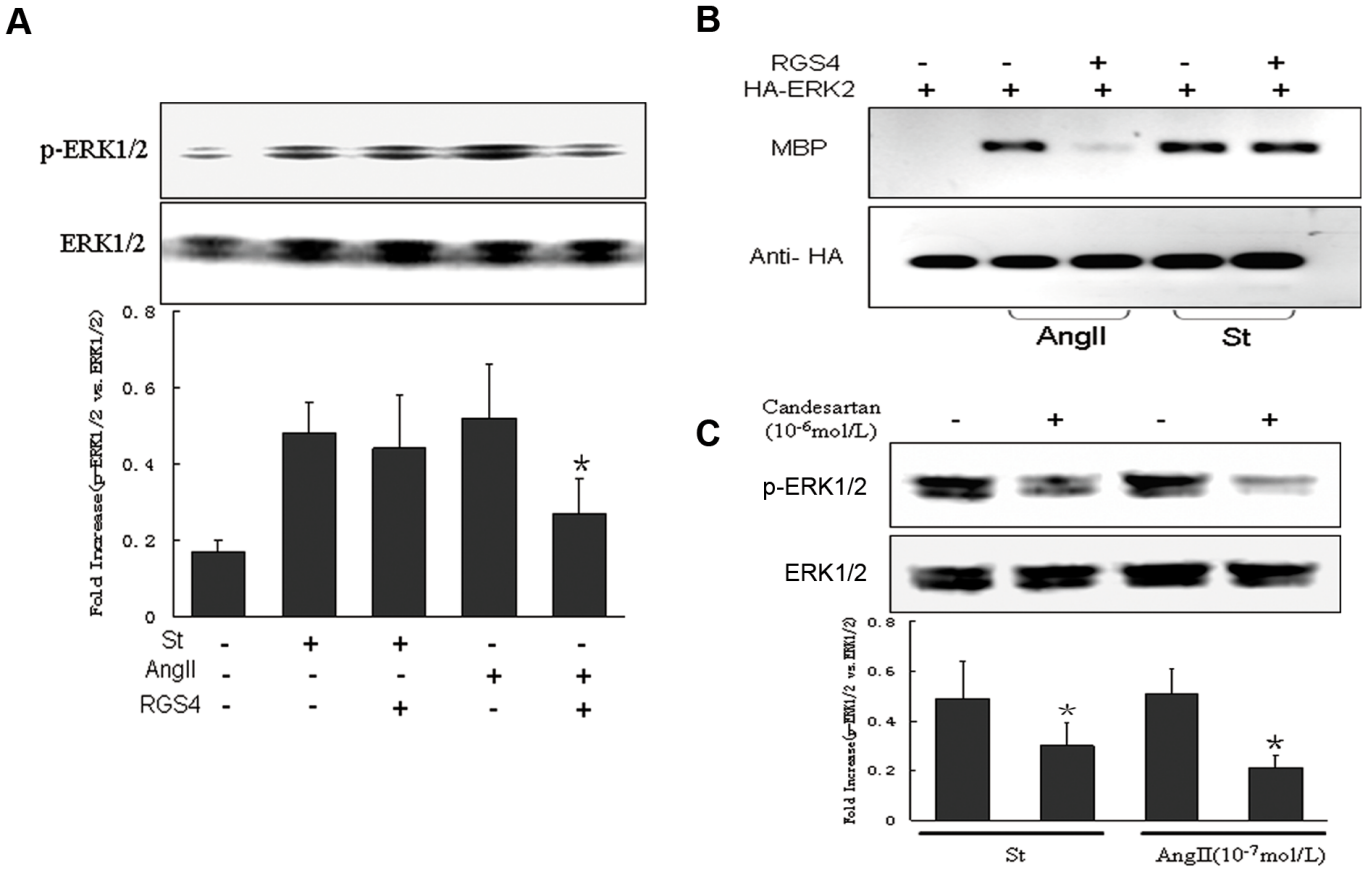
Mechanical stretch induced ERK1/2 signaling via activation of AT1-R, but not affected by G protein inhibition. (**A**) In vitro cultured cardiomyocytes were transfected with or without RGS4 plasmid, and then stimulated by stretch or AngII (10^−7^ mol/L) for 10 min, total proteins were collected and the expressions of phosphorylated ERK1/2 and total ERK1/2 were determined by Western blotting. (**B**) HA-tagged ERK2 was co-transfected with RGS4 plasmid into HEK-293 AT1 expressed cells for 24 h, ERK2 activity (indicated by MBP expression) was detected in cells induced by stretch or AngII, respectively. (**C**) Cardiomyocytes were pretreated with candesartan (10^−6^ mol/L) and then induced by stretch or AngII for 10 min, the expressions of phosphorylated ERK1/2 and total ERK1/2 were determined. * *P*<0.05 vs. stretch-induced group (n = 3 separated experiments).

Increasing evidence implicated that AT1-R could function through a heterotrimeric G-protein coupling or a G-protein-independent mode [18]–[20]. AngII, as the receptor agonist, activated AT1-R via a traditional GTP analog sensitive pathway. Ligand binding to G-protein-coupled receptors (GPCRs) induced GTP binding to the Gα subunit and dissociation from the βγ subunits. Here, we tested the possibility of Gα subunit activation during the process of mechanical stretch-induced AT1-R activation. It was reported that RGS4 negatively regulated Gαq signaling though promoting the hydrolysis of GTP [19], [20]. Thus, the effect of Gαq coupling was attenuated by overexpressing RGS4 in cardiomyocytes. As shown in Figure 1A, Gαq-dependent phosphorylation of ERK1/2 was significantly reduced in AngII (10^−7^ mol/L)-induced cardiomyocytes after overexpressing RGS4. In contrast, RGS4 induced suppression of ERK1/2 was largely prevented by mechanical stretch.

To confirm the role of AT1-R in mechanical stretch evoked signal transduction, we then co-transfected HA-tagged ERK2 with RGS4 in HEK-293-AT1 expressed cells. After transient transfection for 24 h, the cells were stimulated by AngII (10^−7^ mol/L) and mechanical stretch, respectively. Immunoblotting analyses showed that ERK2 phosphorylation (indicated by MBP activity) was markedly attenuated in RGS4 overexpressed cells followed by AngII induction but not by mechanical stretch (Fig. 1B). Of note, both stretch-induced and AngII-stimulated ERK1/2 activation were inhibited by candesartan (10^−6^ mol/L) (Fig. 1C), indicating a critical role of AT1-R in regulating ERK1/2 phosphorylation during mechanical stretch.

### Mechanical stretch-induced ERK1/2 phosphorylation did not depend on Gα coupling pathway

**Figure 2:**
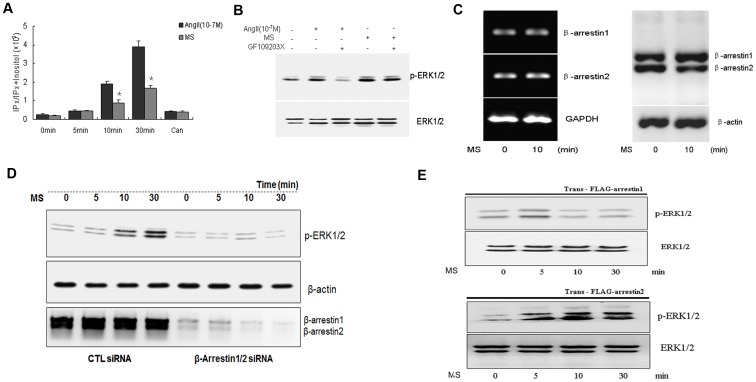
Mechanical stretch preferentially activated a heterotrimeric G protein-independent AT1 signaling pathway. (**A**) In vitro cultured cardiomyocytes were seeded on 24 well plates (1.5×10^5^ cells) and labeled by myo-[^3^H] inositol (1.0 µCi/mL) at 37°C for 24 h. Inositol phosphates (IPx) release was determined at the different indicated time points triggered by AngII (10^−7^ mol/L) or stretch respectively, and including the treatment of candesartan. (**B**) The effect of GF109203X on ERK1/2 phosphorylation stimulated by stretch or AngII (10^−7^ mol/L). (**C**) The endogenous mRNA and protein levels of β-arrestin1 and β-arrestin2 in cardiomyocytes were determined before and after stretch for 10 min. (**D**) Time-dependent ERK1/2 phosphorylation was determined after treatment with β-arrestin1/2 siRNA or scrambled siRNA. (**E**) Mechanical stretch-induced expression of phosphorylated ERK1/2 was determined in a single plasmid (β-arrestin1 or β-arrestin2) transfected HEK-293-AT1 cells after knock-down of endogenous β-arrestin1/2. * *P*<0.05 vs. AngII-induced group (n = 3 separated experiments).

Previous in vivo studies showed that mechanical stretch-induced ERK1/2 activation was significantly attenuated in hearts from β-arrestins KO mice [7], [18]. To explore whether mechanical stretch induced ERK1/2 response via β-arrestin-dependent pathway or Gα-dependent signaling, we first determined the release of inositol 1,4,5-triphosphate (IP_3_) in stretch-induced cardiomyocytes, since CAMP-dependent IP_3_ bioactivity was the major event of GPCRs activation through G*α* protein coupling. As shown in Figure. 2A, IP_3_ release was significantly attenuated in stretching-induced cardiomyocytes compared with that in AngII (10^−7^ mol/L)-induced cardiomyocytes. Both AngII and mechanical stretch mediated IP_3_ release were blocked by candesartan (10^−6^ mol/L), suggesting that AT1 receptor was essential for G*α* protein coupling. In addition, inhibition of PKC with GF109203X, a specific PKC inhibitor, effectively attenuated AngII (10^−7^M) induced ERK1/2 phosphorylation, but the phosphorylation level of ERK1/2 stimulated by stretching was not affected (Fig. 2B). These data suggested that mechanical stretch mediated ERK1/2 signal might not through activating the “classical” G-protein-dependent pathway.

Next, we examined the role of β-arrestin in ERK1/2 activation induced by mechanical stretch. Results showed that both the mRNA and protein levels of β-arrestin1/2 remained unchanged in response to mechanical stretch (Fig. 2C). However, knocking down endogenous β-arrestin1/2 caused a significant decrease in the phosphorylation level of ERK1/2 in response to mechanical stretch (Fig. 2D). We further found that a robust increase of phosphorylated ERK1/2 at 10 min after stretching, which was induced by β-arrestin2 but not by β-arrestin1 in β-arrestin1 or 2 single transfected HEK-293-AT1 cells after knocking down the endogenous β-arrestin1/2 with specific siRNA (Fig. 2E).

### β-arrestin2 regulated ERK1/2 phosphorylation through interacting with the tyrosine kinase Src during mechanical stretch

**Figure 3:**
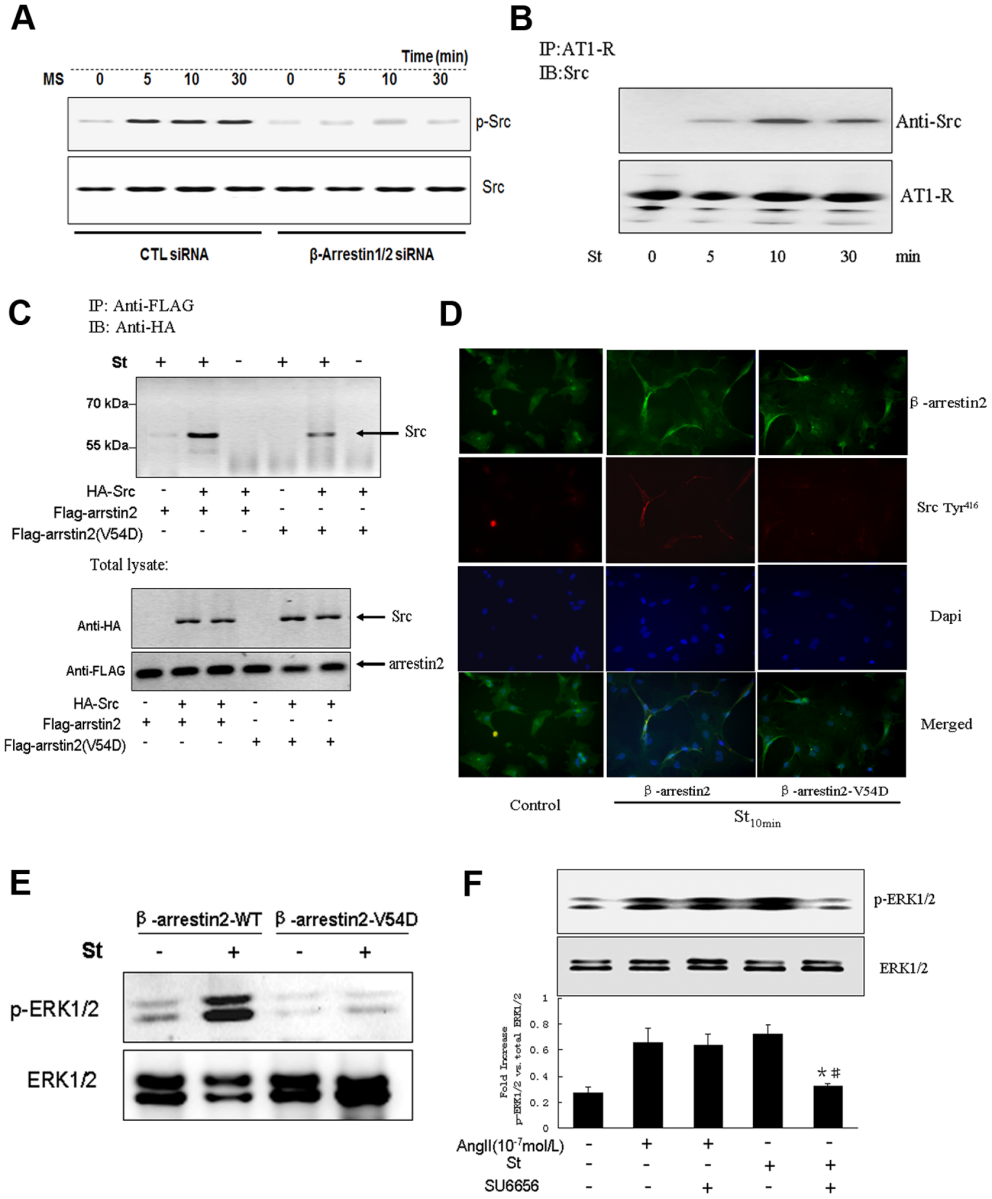
Src/β-arrestin2 signal complex was required for mechanical stretch-mediated AT1-R signaling. (**A**) Western blotting analyses of time-dependent Src phosphorylation after treatment with β-arrestin1/2 siRNA or scrambled siRNA. (**B**) Coimmunoprecipitation analyses of Src and AT1-R in lysates of stretched cardiomyocytes in a time-dependent manner. (**C**) HA-tagged Src and FLAG-tagged β-arrestin2 (or dominant negative β-arrestin2-V54D) were transient transfected into HEK-293-AT1 cells, whole cell extracts were immunoprecipitated with anti-FLAG monoclonal antibody, and the proteins in the immunoprecipitates and in the total lysates were probed by Western blotting using an anti-HA antibody. (**D**) The intercellular location of Src kinase in GFP-tagged β-arrestin2 WT and dominant negative GFP-tagged β-arrestin2-V54D transfected HEK-293-AT1 cells was visualized by immunofluorescence after stretching cells for 10 min. (**E**) The effect of β-arrestin2 WT or β-arrestin2-V54D transfection on mechanical stretch-induced ERK1/2 phosphorylation. (**F**) The effect of SU6656 (5 mmol/L) on ERK1/2 phosphorylation in cardiomyocytes stimulated by mechanical stretch or AngII (10^−7^ mol/L). * *P*<0.05 vs. both AngII and SU6656 treated groups; # *P*<0.05 vs. stretched groups (n = 3 separated experiments).

Above data indicated that β-arrestin2 was critically involved in mechanical stretch-induced ERK1/2 phosphorylation, however, both mRNA and protein levels of β-arrestin2 were not affected by stretching, but the phosphorylation level of Src was suppressed by knocking down of β-arrestin2 (Fig. 3A). Therefore, we assumed that β-arrestin2 might be the scaffold protein that mediated AT1-R-dependent tyrosine kinase phosphorylation. In the Co-IP analysis, we revealed that Src was immunoprecipitated by anti-AT1-R antibody in a time dependent manner (Fig. 3B), suggesting that Src was recruited to the cell membrane and interact with AT1-R during stretching. Our previous observation indicated that one kind of Src family tyrosine kinase was critically involved in mechanical stretch-evoked AT1-R signaling [3], but a direct interaction between Src and β-arrestin2 had not been demonstrated in the model of mechanical stretch. Hence, we co-transfected FLAG-tagged β-arrestin2 and HA-tagged Src into HEK-293-AT1 cells. Afterwards, co-immunoprecipitation assays were conducted in stretched or unstretched HEK-293-AT1 cells. The proteins were collected after stretching for 10 min, and followed by immunoprecipitation of β-arrestin2 with anti-FLAG antibody. We probed the immunoprecipitated sample by Western blotting using anti-HA antibody. The results showed that β-arrestin2 interacted with Src only in stretched cells (Fig. 3C), suggesting that AT1-R mediated recruitment of β-arrestin2 might be important for Src binding and activation. But Src kinase was markedly reduced in sample from cells transfected with dominant negative β-arrestin2-V54D (Fig. 3C). Previous studies reported that β-arrestin, which functioned as a scaffold protein, could recognize and interact with the catalytic domain of Src, by which regulated its kinase activity. Therefore, the attenuated interaction between Src and β-arrestin2-V54D might be due to the lack of binding site for SH2 domain kinase docking [25]. Cell immunofluorescence further confirmed that Src kinase was phosphorylated and recruited to membrane by accumulated β-arrestin2 after mechanical stretch, but this effect was abolished by mutating the Src binding site in β-arrestin2-V54D transfected cardiomyocytes (Fig. 3D). Taken together, these data suggested that mechanical stretch-induced translocation of β-arrestin2 was essential for Src docking and signal transduction. To further investigate the effect of β-arrestin2/Src interaction after stretching on cardiomyocyte hypertrophy, we examined stretch-induced ERK1/2 phosphorylation in both β-arrestin2 and β-arrestin2-V54D transfected cardiomyocytes. The results showed that ERK1/2 phosphorylation was significantly attenuated in cells transfected with β-arrestin2-V54D compared with that in β-arrestin2 transfected cells (Fig. 3E). In addition, mechanical stretch-induced ERK1/2 activation was also inhibited by pretreating cardiomyocytes with SU6656 (10 µmol/L), a selective Src family kinase inhibitor. Of note, the inhibitory effect of SU6656 on ERK1/2 phosphorylation was not observed in cardiomyocytes stimulated by AngII (10^−7^ mol/L) (Fig. 3F). Collectively, these data confirmed that β-arrestin2/Src interaction was uniquely involved in mechanical stretch-mediated ERK1/2 activation.

### Src inhibition attenuated pressure overload-induced myocardial hypertrophy

**Figure 4:**
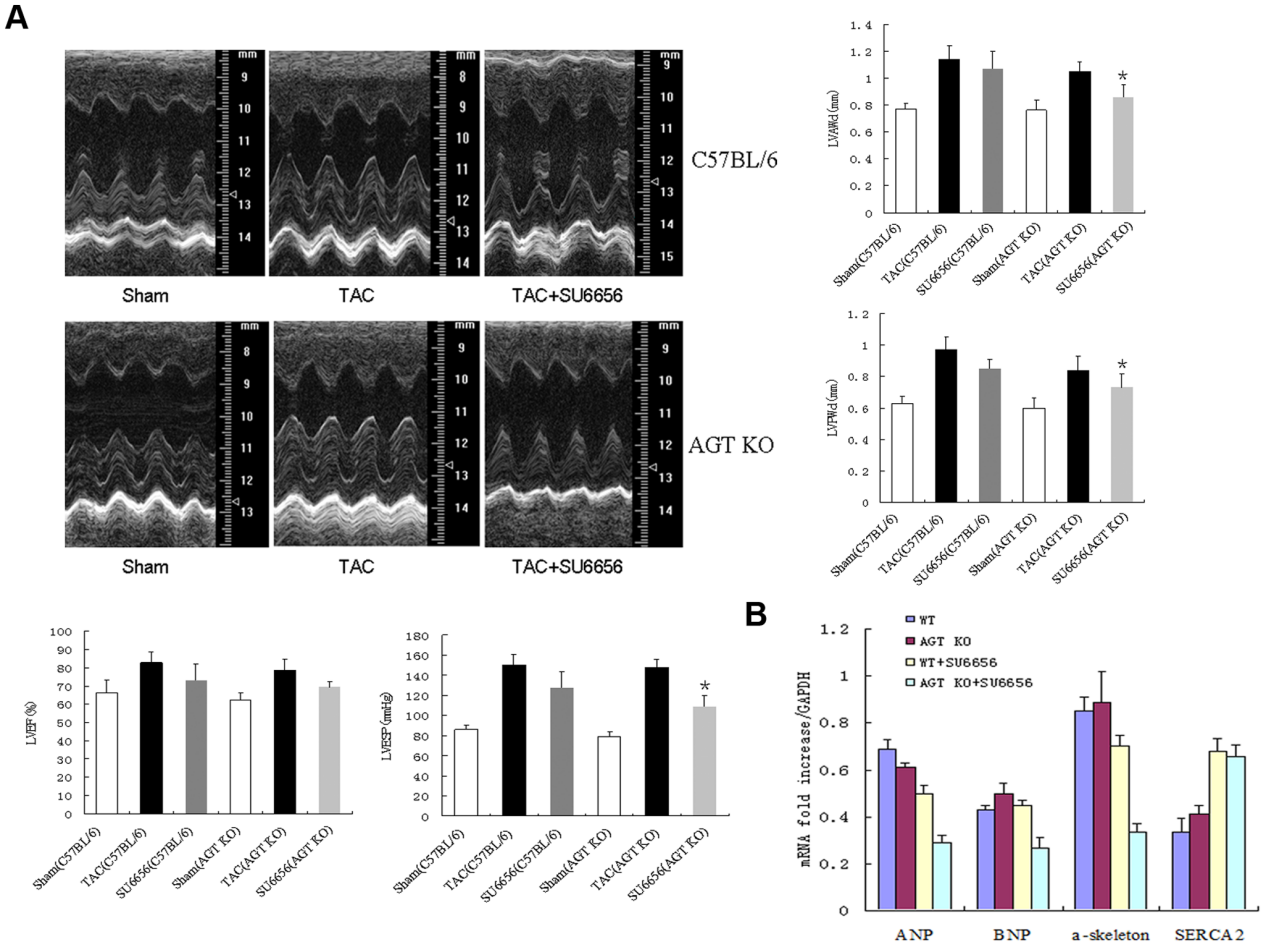
In vivo analyses of the cardiac function by echocardiography and hemodynamic measurements. Both AGT KO mice and the C57BL/6 WT littermates were pretreated with or without Src kinase inhibitor (SU6656), followed by TAC for 2 weeks. (**A**) Quantifications of LVAWd, LVPWd, LVEF and LVESP by representative M-mode tracing and hemodynamic recording from five mice. (**B**) Quantifications of cardiac immediate-early response genes in C57BL/6 mice and AGT KO mice with or without pretreatment of SU6656 (n = 5 separated experiments). * *P*<0.05 vs. saline-treated TAC-operated AGT KO mice.

We then investigated the in vivo consequence of the inhibition of β-arrestin2/Src signaling on pressure overload-induced myocardial hypertrophy in AGT KO mice. At 2 weeks after TAC, echocardiography assessment showed a significant increase of left ventricular anterior wall at end-diastolic (LVAWd) and posterior wall at end-diastolic (LVPWd) thickerness in both C57BL/6 mice and AGT KO mice, accompanied by higher left ventricular ejection fraction (LVEF) and left ventricular end-systolic pressure (LVESP). However, Efficacy of attenuating myocardial hypertrophy by pretreatment with SU6656 was more pronounced in AGT KO mice than in C57BL/6 mice (Fig. 4A). Cardiac reprogramming of specific genes expressions were activated during pressure overload, we therefore determined the immediate-early response genes and fetal genes in the heart after TAC. Pressure overload enhanced the transcriptional levels of ANP, BNP and α-skeleton in AGT KO mice and C57BL/6 littermates, but expression of these re-enhanced genes was significantly lower in AGT KO mice than in C57BL/6 mice after SU6656 treatment, except for sarcoplasmic reticulum Ca^2+^-ATPase (SERCA) (Fig. 4B).

### Effects of different ARBs on AngII and TAC-induced cardiac hypertrophic response in AGT KO mice

**Figure 5:**
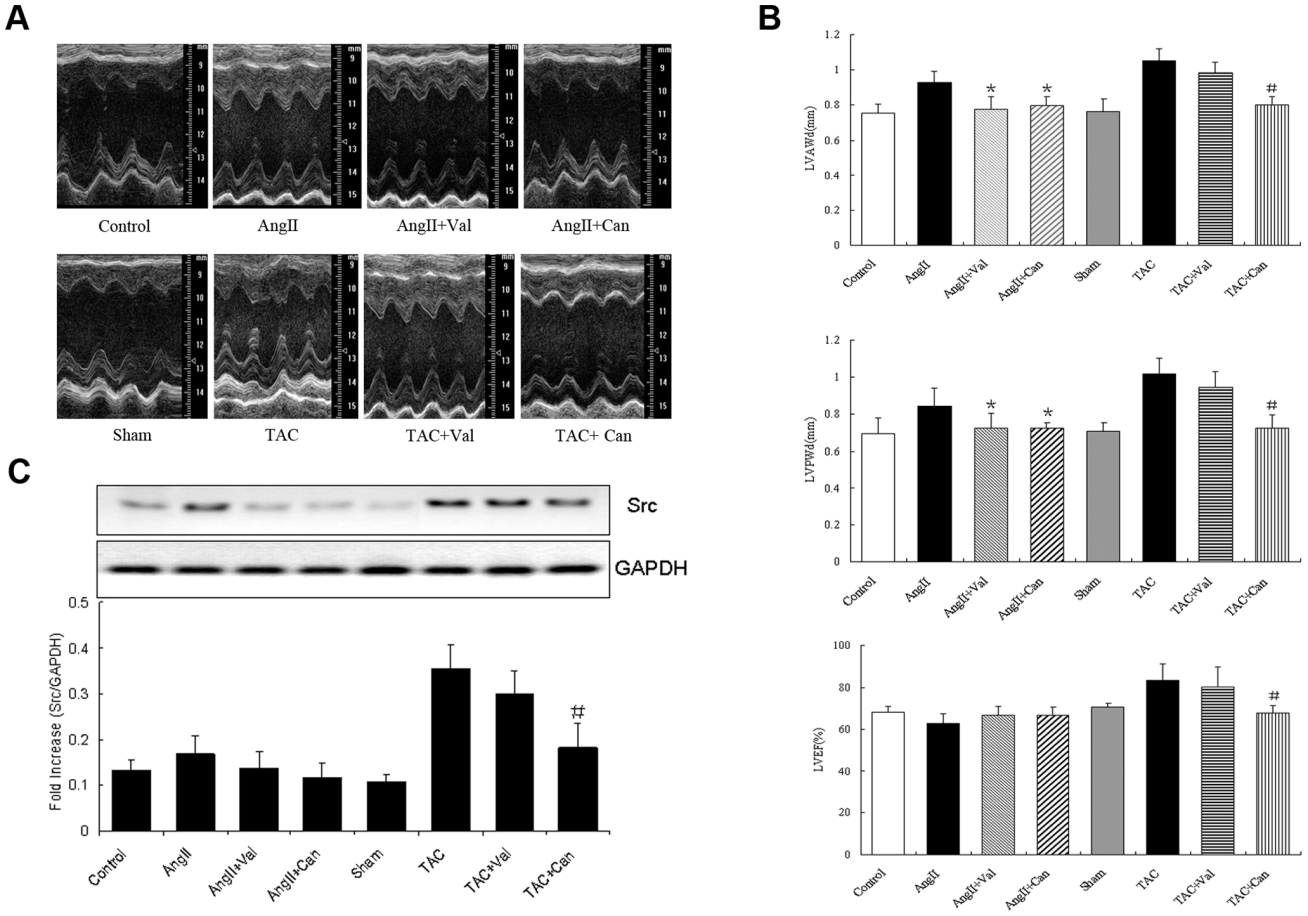
The effects of different ARBs on the cardiac function and Src expression in AGT KO mice. AGT KO mice were induced by AngII or TAC for 2 weeks with or without the pretreatment of valsartan or candesartan. (**A**) Representative M-mode tracings of AGT KO hearts stimulated by AngII (10^−5^ mol/L, top line) or by TAC for 2 weeks (bottom line). (**B**) Representative recording of LVAWd, LVPWd and LVEF in AGT KO mice from each group. (**C**) The expression of Src in myocardium of AGT KO mice from each group was determined. Data were presented as mean ± s.e.m. from five to eight mice. * *P*<0.05 vs. AngII-treated AGT KO mice; # *P*<0.05 vs. TAC-treated AGT KO mice.

We further compared the effects of candesartan and valsartan on myocardial hypertrophic response induced by TAC and AngII stimulation in AGT KO mice. Results showed that both candesartan and valsartan exerted inhibitory impacts on AngII-induced cardiac hypertrophy, but only candesartan effectively reversed TAC-induced hypertrophic response (Fig. 5A–B). Furthermore, Src was weakly upregulated by AngII but robustly enhanced by TAC for 2 weeks, and AngII-induced upregulation of Src was inhibited by both candesartan and valsartan, while TAC-induced Src activation was inhibited only by candesartan (Fig. 5C).

## Discussion

Cardiac hypertrophy is one of the independent risk factors responsible for myocardial injury and remodeling, and such compensatory response may transit to heart failure. Plenty of previous studies have demonstrated that AT1-R is one of the most important receptors mediating cardiac hypertrophic response through multiple signaling pathways [2], [3], [21], [22]. However, the diversity of AT1-R-mediated signaling in response to hypertrophic stimuli in the heart is still not fully understood. We have previously revealed that mechanical stretch can activate AT1-R without the involvement of AngII [3]. In this study, we further confirm a critical role of Src in regulating AT1-R-mediated intercellular signaling transduction stimulated by mechanical stretch.

Immerging evidences indicated that activation model of GPCRs might undergo different phases due to different stimuli. It was generally believed the first phase of AT1-R activation was G-protein coupling dependent, and this process was hypersensitive to ligand binding. G-protein signaling subtype 4 (RGS4), an inhibitory regulator of GPCR signaling through accelerating GTPase activity, was mainly expressed on cardiomyocytes [23], [24]. In this study, intervention of G-protein coupling by overexpressing RGS4 in cardiomyocytes effectively blocked AngII-stimulated ERK1/2 phosphorylation, the indicator of hypertrophic response, but this effect was almost invisible in mechanical stretched cardiomyocytes. These resgults suggested that stretch-mediated AT1-R activation in the absence of the ligand binding might be sensitive to a second phase of G-protein signaling transduction which was independent of G-protein coupling.

Previous studies illustrated that PKC and IP_3_, two important molecules were required for AngII-mediated modulation of myofilament calcium sensitivity and cardiac excitation-contraction coupling [17], [25]. However, IP_3_ was not fully activated in cardiomyocytes by short time effect of mechanical stretch. Interestingly, MS-induced ERK1/2 phosphorylation was not, suppressed by blocking PKC, suggesting a G-protein coupling independent signaling pathway was involved in stretching-induced cardiac hypertrophy. Of note, SU6656, a Src kinase inhibitor, effectively suppressed stretching-induced ERK1/2 activation, indicating a crucial role of Src but not PKC was required for mechanical stretch-mediated AT1 activation and intracellular signaling.

Rakesh K. previously reported that mechanical stretch evoked an abundant increase of ERK1/2 phosphorylation in hearts from WT mice, but not from AT1-R KO or arrestin2 KO mice [7]. It was believed that distinct conformational states of AT1-R might selectively stimulate signaling via G-protein dependent or independent pathway, while β-arrestin–biased agonism might serve as the central mechanism for mechanical stretch-mediated GPCR signaling, In line with this finding, our data suggested that β-arrestin2 not β-arrestin1 was required for stretch-induced ERK1/2 phosphorylation. However, both mRNA and protein levels of β-arrestin2 remained unchanged after mechanical stretch. Instead, stretch triggered β-arrestin2 translocation to the membrane of cardiomyocyte. Previous reports indicated the divergent roles of β-arrestin1 and β-arrestin2 in regulating intercellular signal via translocation to nucleus and cell surface [16], [18], [26], [27]. In this study, we revealed that β-arrestin2 was highly expressed on cell membrane suggesting that mechanical stretch promoted the membrane recruitment of β-arrestin2 and binding with AT1-R. Plenty of studies demonstrated that GPCRs-mediated β-arrestin2 recruitment was essential not only for receptor internalization, but also for signal transduction [18], [26]. It was also proved that tyrosine kinases-dependent signal cascade was crucial for GPCRs mediated ERK1/2 phosphorylation through transactivating EGFR [7], [27], [28]. However, the underlying mechanism was not yet fully clear. Our data demonstrated that AT1-R-mediated accumulation of Src kinase in response to mechanical stretch was β-arrestin2 dependent, dominant negative β-arrestin2 greatly attenuated Src docking and accumulation after stretching. Recruitment of Src to activated GPCRs had multiple functions, such as MAP kinase activation, receptor internalization, and granule release [29], [30]. In our study, Src activation was essential for β-arrestin2-mediated ERK1/2 phosphorylation. Transfection with mutant β-arrestin2 V54D or inhibition of Src by SU6656 in cardiomyocytes effectively suppressed MS-induced ERK1/2 activation. Src-dependent Ras-ERK1/2 pathway was also reported in the activation of AT1a-i2m receptor, which lacking the binding site for G-protein [9], [10]. Therefore, we proposed that β-arrestin2-mediated Src activation might play an important role in G-protein-independent AT1-R activation and subsequent hypertrophic response.

The activation of cellular oncogenes such as c-myc and c-Src were critically involved in the reprogram progress of gene expression during pressure overload-induced myocardial hypertrophy [31]. Here, we also found enhanced that cardiac reprogramming genes were enhanced in the pressure overload model of AGT KO mice, which indicated that AngII binding signal was not essential for regulation of fetal genes reprogramming and hypertrophy induced by mechanical stretch. Instead, inhibition of Src effectively suppressed the transcripts of ANP, BNP and α-skeleton, reversed the ventricular remodeling and hypertrophic response. In this study, we also found that mechanical stress-induced ERK1/2 response could only be suppressed by candesartan, one kind of inverse agonism, but not by valsartan.. Of note, candesartan inhibited the AT1-R activation through binding two domains of Gln257 in TM6 and Thr287 in TM7, both located at the carboxyl of the receptor, thereby stabilized AT1-R in the inactive state [4], [32]. Previous studies suggested that the carboxyl-terminal residues of AT1-R were required for G-protein independent signal transduction and transactivation of EGFR [28], [33]. Mutation of the conserved YIPP motif in the C terminus of AT1-R, resulted in diminished EGFR transactivation and cardiac hypertrophy in Tg-Y319F mice [33]. Because β-arrestin2-dependent Src recruitment was critically involved in GPCRs-mediated EGFR transactivation, the data presented here further supported the notion that the special effect of candesartan on mechanical stretch-induced ERK1/2 phosphorylation was achieved via inhibition of G-protein independent but β-arrestin2 dependent Src recruitment.

**Figure 6:**
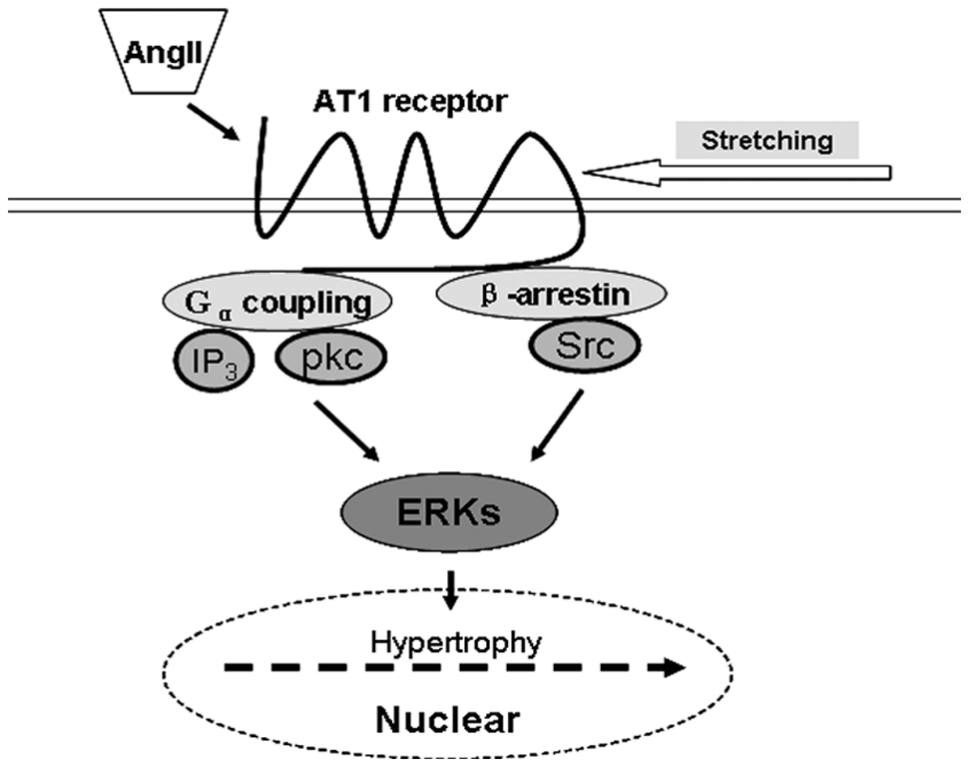
Schema for the mechanism of cardiac hypertrophy via AT1-R-dependent signaling pathway induced by AngII or mediated by mechanical stretch, respectively.

In summary, our study presented the divergent mechanisms for mechanical stretch- and AngII-mediated β-arrestin2-dependent and G-protein coupling-dependent signaling pathway in cardiac hypertrophy. Mechanical stretch-induced conformational switch of AT1-R might be different from that stimulated by AngII, thus resulted in different signal transduction (Fig. 6). The uncovering of β-arrestin2/Src-mediated ERK1/2 phosphorylation in response to mechanical stretch might be crucial for the establishment of pressure overload-induced cardiac hypertrophy, and clarification of this novel mechanism might be helpful for the development of more potent inverse agonist for AT1-R.
